# Prevalence of mild cognitive impairment in community-dwelling Chinese populations aged over 55 years: a meta-analysis and systematic review

**DOI:** 10.1186/s12877-020-01948-3

**Published:** 2021-01-06

**Authors:** Yuan Lu, Chaojie Liu, Dehua Yu, Sally Fawkes, Jia Ma, Min Zhang, Chunbo Li

**Affiliations:** 1 Department of General Practice, Yangpu hospital, Tongji University School of Medicine, Shanghai, 200090 China; 2 School of Psychology and Public Health, La Trobe University, Melbourne, VIC 3086 Australia; 3 Academic Department of General Practice, Yangpu hospital, Tongji University School of Medicine, Shanghai, 200090 China; 4 Shanghai General Practice and Community Health Development Research Center, 200090, Shanghai, China; 5 Clinical Research Center, Shanghai Mental Health Center, Shanghai Jiao Tong University School of Medicine, Shangha, China

**Keywords:** Mild cognitive impairment, Prevalence, Systematic review, Meta-analysis

## Abstract

**Background:**

Mild cognitive impairment (MCI) is an intermediate phase between normal cognitive ageing and overt dementia, with amnesic MCI (aMCI) being the dominant subtype. This study aims to synthesise the prevalence results of MCI and aMCI in community-dwelling populations in China through a meta-analysis and systematic review.

**Methods:**

The study followed the Preferred Reporting Items for Systematic reviews and Meta-Analyses (PRISMA) protocol. English and Chinese studies published before 1 March 2020 were searched from ten electronic bibliographic databases. Two reviewers screened for relevance of the studies against the pre-defined inclusion and exclusion criteria and assessed the quality of the included studies using the Risk of Bias Tool independently. A random-effect model was adopted to estimate the prevalence of MCI and aMCI, followed by sub-group analyses and meta-regression. Sensitivity and publication bias tests were performed to verify the robustness of the meta-analyses.

**Results:**

A total of 41 studies with 112,632 participants were included in the meta-analyses. The Chinese community-dwelling populations over 55 years old had a pooled prevalence of 12.2% [95% confidence interval (CI): 10.6, 14.2%] for MCI and 10.9% [95% CI, 7.7, 15.4%] for aMCI, respectively. The prevalence of MCI increased with age. The American Psychiatric Association’s Diagnostic tool (DSM-IV) generated the highest MCI prevalence (13.5%), followed by the Petersen criteria (12.9%), and the National Institute on Aging Alzheimer’s Association (NIA-AA) criteria (10.3%). Women, rural residents, and those who lived alone and had low levels of education had higher MCI prevalence than others.

**Conclusion:**

Higher MCI prevalence was identified in community-dwelling older adult populations in China compared with some other countries, possibly due to more broadened criteria being adopted for confirming the diagnosis. The study shows that aMCI accounts for 66.5% of MCI, which is consistent with findings of studies undertaken elsewhere.

**Systematic review registration number:**

PROSPERO CRD42019134686.

**Supplementary Information:**

The online version contains supplementary material available at 10.1186/s12877-020-01948-3.

## Background

The World Alzheimer Report 2016 [1] estimated that dementia is the third most serious health problem following cancer and cardio-cerebrovascular diseases, and costs the global economy around 315 billon US dollars annually. Like many other diseases, most of the burden of dementia is experienced by low- and middle-income countries [2]. China, as the most populated middle-income country, has attracted the greatest burden of dementia. About one quarter of people with a dementia diagnosis live in China [3]. The dementia-associated disability and care burden in China is projected to be as high as US$250 billion in 2020, which accounts for nearly one fifth of the global costs associated with dementia [4].

Early intervention measures are considered to be the most cost-effective for managing dementia due to a lack of an effective treatment regimen [5]. Mild cognitive impairment (MCI) has been conceptualised as an intermediate phase between normal cognitive ageing and overt dementia [6]. MCI is a neurological disorder in older adults characterised by slight but noticeable deficits in memory and/or other thinking skills with minimal impacts on daily living functioning [7]. Some researchers argue that MCI represents an early stage of dementia [8], with a tendency of progressing into clinically diagnosed dementia at an annual rate around 30% [9] and a lifetime rate of 60–90% [10].

MCI can be subcategorised into amnesic MCI (aMCI) and non-amnesic (naMCI). Memory loss is the predominant symptom of aMCI compared with naMCI which involves impairment in thinking skills other than memory [11]. Individuals with aMCI tend to progress into Alzheimer’s disease (AD); however, naMCI seems to represent a prodromal phase of frontotemporal dementia and dementia with Lewy bodies. Both aMCI and naMCI can lead to vascular dementia [12].

Internationally, extensive studies have been undertaken to determine the prevalence of MCI, generating great variations in results. A systematic review published in 2012 reported a prevalence of MCI ranging from 0.5 to 42% in different countries and populations [13]. Recent studies in the US [14], Spain [15], Brazil [16], Saudi Arabia [17], and Japan [18] reported a range of MCI prevalence between 6.5 and 38.6%. Significant variations in reported prevalence of MCI also exist within China. The Dementia Research Group reported a MCI prevalence of 0.8% in China [19], compared with 20.8% reported by the Chinese National Centre for Prevention and Control of Chronic and Non-communicable Diseases [20].

This study aims to determine the prevalence of MCI (including its subtypes) in community-dwelling older adults in China through a meta-analysis and systematic review. The study addresses several limitations of the existing systematic reviews [21, 22]. First, there is a need to carefully assess the representativeness of study samples. Inclusion of studies involving participants with certain special characteristics can seriously overestimate or underestimate the prevalence of MCI. For example, a study reported extremely high prevalence of MCI (74.23%) in retired cadres, most in a very senior age [23]. By contrast, another study involving a high proportion of participants younger than 60 years reported only 2.4% of MCI [24]. Second, diagnostic criteria need to be considered in synthesising results. Applying different diagnostic tools and criteria is likely to lead to different results [25]. Many studies have failed to report specified criteria for confirmation of MCI [26]. Third, discrepancies in findings across study settings are common and they should not be mixed in synthesising analyses. MCI prevalence is usually higher in institutional settings than in communities [27]. To overcome the above-mentioned shortfalls, this study performed a series of subgroup analyses. To the best of our knowledge, no meta-analysis on aMCI prevalence in China has been reported. Findings of this study, especially those of the subgroup analyses, can provide a solid foundation for estimating MCI prevalence in community residents with different characteristics. This data is critical for planning preventive services in community settings.

## Methods

We followed the Preferred Reporting Items for Systematic Reviews and Meta-Analyses (PRISMA) protocol (supplementary file 1), which delineates a four-phase flow diagram and a 27-item checklist (www.prisma-statement.org). The protocol of this systematic review was registered on PROSPERO and is available in full on the website https://www.crd.york.ac.uk/PROSPERO/display_record.php? ID=CRD42019134686.

### Search strategy

The well-established databases in English (Google Scholar, PubMed, Web of Science, Embase, CINAHL, PsycINFO) and Chinese languages (CQVIP, Wangfang, CNKI, Sinomed) were searched. All of the databases were searched from their inception to the 1^st^ of March 2020, using a combination of the following searching terms: (“mild cognitive impairment” or “cognitive dysfunction” or “early dementia”) and (epidemiology, prevalence, rate, occurrence) (details provided in supplementary file 2). Hand searches were also performed to identify related papers through reference lists of the identified studies. A research librarian was consulted in developing the search strategy. The search results were exported to Endnote X9 (Thomson Reuters).

### Data extraction

A total of 2136 studies were identified after deletion of duplications. Two reviewers (MZ and JM) screened the titles and abstracts of the articles and identified those that met the inclusion and exclusion criteria. The included articles had to fall into the category of original studies, including both population-based cross-sectional and longitudinal studies in community-based samples, with prevalence of MCI and/or aMCI as a primary study objective. The study samples were representative of community-dwelling older adults as indicated by the sampling strategy and did not include those admitted to long-term care facilities. MCI cases were identified using a MCI screening strategy followed by diagnostic confirmation. Since the pathobiological process in the human brain happens decades before the onset of dementia [28] and MCI screening may reasonably start at the age of 55 years, studies involving participants aged over 55 years were deemed eligible. There is a lack of consensus about when MCI screening should be started. Empirical evidence shows that the prevalence of MCI increases with age [23, 24]. This study included participants ≥55 years simply because there were no eligible MCI studies involving participants under 55 years old. In China, women and those engaging in labor-intensive jobs usually retire at the age of 55 years and are eligible for some preventive care packages delivered by community health services. These may include community MCI screening. Studies with a sample restricted to those with special characteristics such as disease condition (e.g. Parkinson disease, depression, stroke), occupation, internal migration, insurance, and literacy were excluded. Full texts of the eligible articles (*n* = 172) were then further assessed against the inclusion and exclusion criteria, and another 127 articles were excluded for failing to meet the inclusion criteria.

**Fig. 1:**
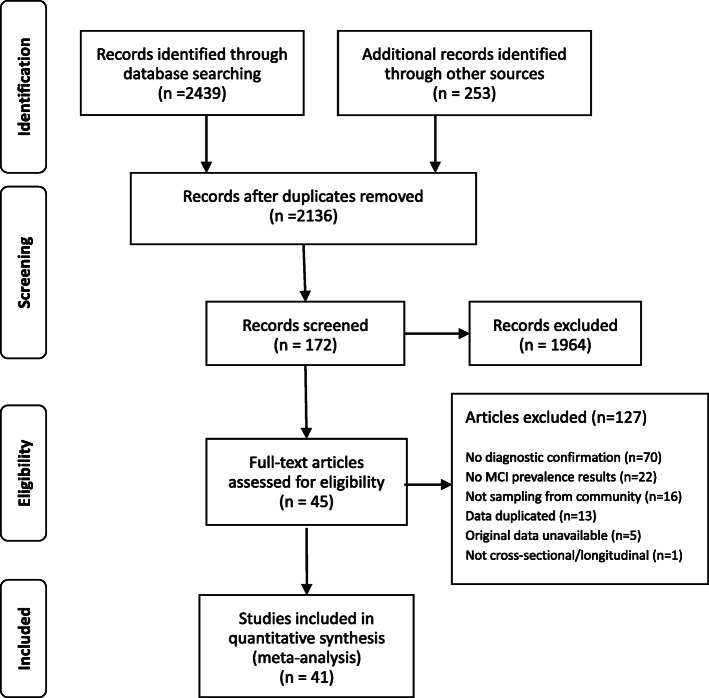
the flowchart on the stages of including the studies in the systematic reviewed and meta-analysis (PRISMA 2009)

Two reviewers (MZ and JM) assessed the quality of the 45 studies that met the inclusion criteria by extracting key elements from the full texts into the Risk of Bias Tool [29]. The Risk of Bias Tool examines four aspects of external validity (target population representation, sample representation, random sampling, non-response bias), five aspects of internal validity (data collection proxy, acceptable case definition, instrument validity and reliability, data collection mode, appropriate parameter), and the overall risk of bias of the studies. This tool was designed for assessing bias in epidemiological surveys. The grading of the assessed aspects adopted the Cochrane Grades of Recommendation, Assessment, Development, and Evaluation (GRADE) scheme [30]. Each assessed aspect was given a rating of “low”, “medium” or “high” risk of bias. Discrepancies between the two reviewers, if occurred, were resolved through discussions moderated by a third researcher. This resulted in a final sample of 41 studies without a high risk of bias for the final meta-analysis (Fig. 1).

Apart from the prevalence of MCI (including aMCI and naMCI), data in relation to study setting (urban/rural), demographic characteristics (age, sex, educational attainments, living status) of participants, study period, screening tools, and diagnostic confirmation methods for each of the included studies were extracted. Empirical evidence shows that the prevalence of MCI/aMCI is likely to vary by these factors [31].

### Statistical analysis

MCI prevalence was the primary outcome of this meta-analysis. We synthesised the results for MCI in general as well as for aMCI specifically.

Publication bias of the included studies was assessed through visual symmetry of the funnel plots and Egger’s tests [32]. A *p* value lower than 0.05 indicates an absence of publication bias.

We performed heterogeneity analyses to determine the model used for meta-analyses. The I^2^ value was calculated and tested with Cochran Q tests. According to Cochrane Reviews [33], an I^2^ value of 0% indicates no observed heterogeneity, while a value greater than 25, 50 and 75% indicates low, moderate, and high levels of heterogeneity, respectively. We chose a random effect model for meta-analyses and adopted sensitivity tests, meta-regression test and subgroup analyses strategies to handle high heterogeneity as suggested by Lipsey and Wilson [34]. The robustness of the meta-analyses was examined in sensitivity tests through sequentially removing each included study. The studies that deviated significantly from the others were excluded in the pooled results. Meta-regression was used to investigate available contributing factors on the heterogeneity. Subgroup meta-analyses were conducted whenever possible.

All statistical analyses were performed using Stata version 12.0 (Stata Corp, College Station, TX, USA). A two-sided *p* < 0.05 was considered statistically significant. We also reported 95% confidence intervals (CIs) for the results.

## Results

### Characteristics of included studies

The 41 eligible studies [19, 20, 24, 35–72] involved 112,632 study participants, with MCI prevalence ranging from 1.21 to 33.03%. The studies were conducted between 1998 and 2020. More than half (58%) of the studies restricted participants from the age of over 60 years and 75% of the included studies had a sample size over 1000 participants. Most studies were cross-sectional, except for 7 longitudinal studies [39, 43, 46, 59, 61, 63, 66]. Sex composition varied across the studies, with women comprising 25.74 to 66.89% of study participants. About 30 neuropsychological test tools were used in the included studies of this systematic review. Of those tools, some test comprehensive cognitive function, such as CAMCOG (Cambridge Cognitive Examination) [73], CCAS (Chinese Cognitive Ability Scale) [74], CSI-D (Community Screening Instrument for Dementia) [75], MMSE (Mini-Mental State Examination) [76], MoCA (the Montreal Cognitive Assessment) [77], NPI-Q (Neuropsychiatric Inventory Questionnaire) [78], OCST-E (Quick Cognitive Screening Scale) [79], SCID (Structured Clinical Interview for DSM-IV) [80], WAIS (Wechsler Adult Intelligence Scale) [81], WHO-BCAI (World Health Organization-Battery of Cognitive Assessment Instrument) [82] and WHODAS-12 (12-item WHO Disability Assessment Schedule) [83], while others were adopted to test five cognitive domains: memory assessed by ALT (Associative Learning Test) [84], IMCT (Information-Memory-Concentration test) [85] and WMS-R (Wechsler Memory Scale-Revised) [86]; attention assessed by SDMT (Symbol Digit Modalities Test) [87] and STMT (Semantic Trail Making Test) [88]; vision tested by CDT (Clock drawing Test) [89] and ROCFR (Rey-Osterrieth Complex Figure Recall tests) [90]; language tested by AVLT (Auditory Verbal Learning Test) [91] and VFT (Verbal Fluency Test) [92]; and executive ability tested by ADL (Activity Daily Living) [93] and FAQ (Functional Activities Questionnaire) [94]. CDR (Clinical Dementia Rating Scale) [95], CESD (The Center for Epidemiologic Studies Depression Scale) [96], DS (Digit Span) [97], HAMD (Hamilton Depression Rating Scale) [98], GDS (Global Deterioration Scale) [99], GMS (Geriatric Mental State) [100], SAS (Self-Rating Anxiety Scale) [101], and HIS (Hachinski Ischemic Index) [102] were used to exclude dementia and other mental disorder. MMSE [76] and the MoCA [77] scales were the predominant tools for MCI screening, supplemented by ADL [93] to test daily living function and other neurological tests such as CDR [95] to exclude dementia. The majority of the studies (*N* = 25) adopted Petersen’s criteria for confirmation of diagnosis of MCI and aMCI, followed by the Diagnostic and the Statistical Manual of Mental Disorder 4th edition (DSM-IV) [80] developed by the American Psychiatric Association and the National Institute on Aging Alzheimer’s Association (NIA-AA) [103] criteria (Table 1).

**Table 1:** Characteristics of studies (*n* = 41) included for meta-analysis

First Author	Study period	Sample size	Age (years)	Sex (% women)	Study location	Diagnostic criteria	Neurological tests	MCI prevalence (95%CI)
Chen ND	2012	465	≥60	30.75%	Jiangsu	Petersen	ADL [93], CDT [89], SAS [101]	10.75% (8.25–13.9%)
Ding D	2011	2985	≥60	54.22%	Shanghai	Petersen	ADL [93], SAS [101], CDR [95], HAMD [98], MMSE [76]	20.10% (18.73–21.61%)
Guo GY	2013	940	≥60	56.81%	Hebei	Petersen	MoCA [77]	14.47% (12.36–16.86%)
Guo X	2011	1367	≥60	50.40%	Hunan	DSM-IV	CDR [95], GDS [99], HIS [102], IMCT [85], MMSE [76]	10.17% (8.68–11.88%)
Hai S	2007	202	≥80	25.74%	Sichuan	Petersen	ADL [93], CAMCOG [73], GDS [99], MMSE [76]	30.2% (24.28–36.85%)
He L	2014	842	≥60	48.81%	Jiangxi	DSM-IV	ADL [93], CDR [95], GDS [99], HIS [102], MoCA [77]	13.42% (11.28–15.89%)
Hu R	2009	5887	≥55	56.38%	Mongolia	DSM-IV	ADL [93], CDR [95], HIS [102], HAMD [98], MMSE [76], MoCA [77]	20.60% (19.59–21.66%)
Huang R	2002	4697	≥60	58.85%	Guangzhou	Petersen	ADL [93], CDR [95], CESD [96], HAMD [98], MMSE [76]	5.47% (4.86–6.16%)
Jia J	2009	10,276	≥65	N/A	National	Petersen	AVLT, CDR [95], CDT [89], CESD [96], FAQ [94], HIS, MoCA [77], MMSE [76], STMT [88], VFT [92], AVLT [91]	20.8% (20.02–21.59%)
Jiang LJ	2016	895	≥60	51.06%	Jilin	NIA-AA	ADL [93], CDR [95], GDS [99], HIS [102], MMSE [76], MoCA [77]	5.36% (4.07–7.04%)
Lao ML	2010	7665	≥55	54.22%	Hainan	Petersen	ADL [93], GDS [99], MMSE [76]	4.25% (3.82–4.73%)
Li CP	2014	1971	≥60	62.61%	Shandong	DSM-IV	ADL [93], CDR [95], GDS [99], HIS [102], MMSE [76]	33.03% (30.99–35.14%)
Li W	2019	3246	≥60	N/A	Shanghai	Petersen	ALT [84], AVLT [91], DS [97], MMSE [76], MoCA [77], NPI-Q [78], VFT [92], WAIS [81]	17.07%^*^ (15.79–18.42%)
Li X	2013	1020	≥55	63.33%	Beijing	Petersen	AVLT [91], CDT [89], MMSE [76], ROCFR [90], SDMT [87], STMT [88]	15.69% (13.58–18.05%)
Liao B	2012	399	≥60	53.63%	Jiangxi	Petersen	ADL [93], HIS [102], MoCA [77]	10.28% (7.67–13.64%)
Liu H	2018	1796	≥60	53.95%	Shanghai	DSM-IV	ADL [93], GDS [99], HIS [102], MoCA [77]	17.65% (15.96–19.48%)
Ma F	2016	5067	≥65	57.80%	Tianjin	Petersen	ADL [93], MMSE [76], WAIS [81]	11.33% (10.48–12.23%)
Meng WQ	2009	5452	≥55	53.62%	Inner Mongolia	Petersen	ADL [93], MMSE [76]	22.50% (21.16–23.37%)
Pan ZD	2012	300	≥60	57.14%	Shanghai	Petersen	ADL [93], CDR [95], GDS [99], HIS [102], MMSE [76], MoCA [77]	22.33% (17.99–27.38%)
Qin HY	2012	4086	≥55	65.00%	Shanghai	Petersen	ADL [93], CDR [95], MMSE [76]	14.98% (13.92–16.11%)
Qiu CJ	2001	3910	≥55	50.82%	Chengdu	Petersen	CDR [95], CESD [96], MMSE [76]	2.35% (1.92–2.88%)
Rao DP	2009	2111	≥65	59.50%	Guangzhou	Petersen	ADL [93], CDR [95], GDS [99], MMSE [76], MoCA [77]	14.16% (12.74–15.72%)
Ren CF	2011	946	≥60	49.26%	Jiangxi	DSM-IV	ADL [93], CDR [95], GDS [99], HIS [102], MoCA [77]	10.47% (8.67–12.58%)
Song XZ	2011	2279	≥60	51.21%	Guangzhou	Petersen	ADL [93], CDR [95], GDS [99], HIS [102], HAMD [98], MMSE [76]	7.33% (6.33–8.47%)
Sosa AL	2007	2014	≥65	63.33%	National	DSM-IV	CSI-D [75], GMS [100], NPI-Q [78], WHODAS-12^[83]^	7.99%^*^ (6.89–9.26%)
Su C	2011	341	≥60	52.49%	Guangzhou	Petersen	ADL [93], CDR [95], GDS [99], WHO-BCAI [82], MMSE [76], MoCA [77]	12.32% (9.24–16.23%)
Sun Y	2013	10,432	≥65	52.32%	Taiwan	NIA-AA	ADL [93], CDR [95], MMSE [76]	19.64% (18.89–20.41%)
Tang MN	1998	5385	≥55	N/A	Chengdu	DSM-III	ADL [93], CDR [95], CESD [96], HIS [102], HAMD [98], MMSE [76]	1.21% (0.95–1.54%)
Tang Z	2004	1865	≥60	51.90%	Beijing	Petersen	ADL, CDR [95], CESD [96], MMSE [76]	11.64% (10.26–13.17%)
Wang T	2012	1005	≥60	N/A	Shanghai	DSM-IV	ADL [93], AVLT [91], CDR [95], DS [97], GDS [99], HIS [102], MMSE [76], MoCA [77], WMS-R [86]	22.29%* (19.82–24.96%)
Wang TT	2017	1781	≥60	60.47%	Chongqing	Petersen	ADL [93], GDS, MMSE [76]	11.73% (10.32–13.31%)
Wang YP	2009	6152	≥65	N/A	Shanxi	DSM-IV	CDR [95], MMSE [76], WHO-BCAI [82]	9.75% (9.04–10.52%)
Wang ZZ	2013	689	≥55	62.70%	Ningxia	Chinese Dementia guideline	ADL [93], GDS [99], MMSE [76]	18.29% (15.58–21.35%)
Wu Y	2014	1846	≥60	53.36%	Jiangsu	Petersen	CCAS [74], CDR [95], HAMD [98], MMSE [76], QCST-E [79]	17.17% (15.52–18.96%)
Xiao SF	2016	1068	≥60	N/A	Shanghai	Petersen	AVLT [91], MMSE [76], MoCA [77], WMS-R [86], WHO-BCAI [82]	25.00% (22.50–27.68%)
Xu SJ	2011	2426	≥60	60.68%	Hebei	Petersen	ADL [93], GDS [99], MMSE [76], MoCA [77], SAS [101]	21.68% (20.09–23.37%)
Yin LY	2009	1011	≥65	59.45%	Hebei	Petersen	CDR [95], CESD [96], FAQ, GDS [99], MMSE [76], MoCA [77]	6.63% (5.25–8.33%)
Yuan J	2010	3311	≥60	66.89%	Shanghai	Petersen	HIS [102], SCID-I/P [80]	19.06% (17.76–20.43%)
Zhang XQ	2012	1764	≥60	55.95%	Changsha	Petersen	ADL [93], CDR [95], GDS, MMSE [76], MoCA [77]	16.27% (14.62–18.07%)
Zhou DS	2010	1227	≥60	56.32%	Zhejiang	DSM-IV	CDR [95], CESD [96], GDS [99], HIS [102], IMCT [85], MMSE [76]	8.72% (7.27–10.43%)
Zhu XQ	2008	1511	≥60	54.60%	Xinjiang	DSM-IV	CDR [95], GD S [99], HIS [102], HAMD [98], MMSE [76]	9.79% (8.40–11.40%)

Note: * aMCI prevalence

### Publication bias

Most of the included studies were rated as having a moderate risk of bias, except for the four studies [104–107], which have a high risk of bias and were excluded from the final meta-analysis (Table 2).

**Table 2:** Risk of bias of included studies (*n* = 45)

No.	Study	External validity	Internal validity	Overall
1	Chen ND, 2012	Moderate Risk	Moderate Risk	Moderate Risk
2	Ding D, 2015	Low Risk	Low Risk	Low Risk
3	Guo GY, 2013	Moderate Risk	Moderate Risk	Moderate Risk
4	Guo XY, 2013	Moderate Risk	Moderate Risk	Moderate Risk
5	Hai S, 2011	Moderate Risk	Moderate Risk	Moderate Risk
6	He L, 2015	Low Risk	Moderate Risk	Moderate Risk
7	Hu R, 2012	Moderate Risk	Moderate Risk	Moderate Risk
8	Huang R, 2008	Low Risk	Low Risk	Low Risk
9	JIA J, 2013	Low Risk	Low Risk	Low Risk
10	Jiang LJ, 2017	Moderate Risk	Moderate Risk	Moderate Risk
11	Lao ML, 2011	Moderate Risk	Moderate Risk	Moderate Risk
12	Li CP, 2014	low Risk	Moderate Risk	Moderate Risk
13	Li X, 2013	Moderate Risk	Moderate Risk	Moderate Risk
14	Li W, 2020	low Risk	Low Risk	Low Risk
15	Liao B, 2012	Moderate Risk	Moderate Risk	Moderate Risk
16	Liu H, 2018	low Risk	Moderate Risk	Moderate Risk
17	Ma F, 2016	Low Risk	Low Risk	Low Risk
18	Meng WQ, 2010	Moderate Risk	Moderate Risk	Moderate Risk
19	Pan HY, 2012	Moderate Risk	High Risk	High Risk
20	Pan ZD, 2012	Low Risk	Moderate Risk	Moderate Risk
21	Peng Z, 2019	Moderate Risk	High Risk	High Risk
22	Qin HY, 2014	Low Risk	Low Risk	Low Risk
23	Qiu CJ, 2003	Moderate Risk	Low Risk	Moderate Risk
24	Rao D, 2018	Low Risk	Low Risk	Low Risk
25	Ren CF, 2013	Moderate Risk	Low Risk	Moderate Risk
26	Song XZ, 2012	Low Risk	Moderate Risk	Moderate Risk
27	Sosa AL, 2012	Moderate Risk	Moderate Risk	Moderate Risk
28	Su C, 2013	Moderate Risk	Moderate Risk	Moderate Risk
29	Sun Y, 2014	Low Risk	Moderate Risk	Moderate Risk
30	Tang Z, 2007	Low Risk	Moderate Risk	Moderate Risk
31	Tang MN, 2000	Low Risk	Moderate Risk	Moderate Risk
32	Wang T, 2017	Low Risk	Moderate Risk	Moderate Risk
33	Wang TT, 2017	Low Risk	Low Risk	Low Risk
34	Wang YP, 2011	Moderate Risk	Moderate Risk	Moderate Risk
35	Wang ZZ, 2013	Moderate Risk	Moderate Risk	Moderate Risk
36	Wu L, 2016	Moderate Risk	High Risk	High Risk
37	Wu Y, 2017	Low Risk	Moderate Risk	Moderate Risk
38	Xiao SF, 2016	Low Risk	Low Risk	Low Risk
39	Xu SJ, 2014	Low Risk	Moderate Risk	Moderate Risk
40	Yin LY, 2010	Low Risk	Moderate Risk	Moderate Risk
41	Yuan J, 2013	Moderate Risk	Moderate Risk	Moderate Risk
42	Zhang XQ, 2014	Low Risk	Low Risk	Low Risk
43	Zhong SY, 2018	Moderate Risk	High Risk	High Risk
44	Zhou DS, 2011	Moderate Risk	Low Risk	Moderate Risk
45	Zhu XQ, 2009	Moderate Risk	Low Risk	Moderate Risk

### Robust tests for pooled results

High levels of heterogeneity were found (I^2^ > 75%) across the 41 included studies. Of the 38 studies reporting MCI prevalence, three [39, 45, 59] showed significant deviation from the others both in sensitivity tests and visual funnel asymmetry. The Egger’s and Begg’s tests also revealed significant publication bias in the studies (*β* = 0.002, *p* < 0.01). Cohort effects could explain 20.75% of heterogeneity from meta-regression test. Further subgroup analyses on MCI prevalence were warranted as no significant associations (*p*>0.05) between the prevalence of MCI and other two potential bias factors were found in the meta-regression analyses (Table 3).

**Table 3:** Meta-regression analyses result

lnhr	Coef.	Std. Err.	t	P > |t|	[95% Conf. Interval]	
Age	.2187126	.1562825	1.40	0.171	−.0988917	.5363169
Study period	.3282157	.1214176	2.70	0.011	.0814654	.574966
Diagnostic criteria	−.1795994	.1203679	−1.49	0.145	−.4242164	.0650176
Constant	−3.146882	.5048756	−6.23	0.000	−4.172913	−2.120851

Of the 8 studies [19, 20, 36, 46, 47, 50, 54, 61] reporting aMCI prevalence, no study showed significant deviation from the others in sensitivity tests. The Egger’s and Begg’s tests revealed no significant publication bias either (*β* = 1.0, *p* = 0.902).

### Prevalence of MCI and aMCI – results of meta-analyses

**Fig. 2:**
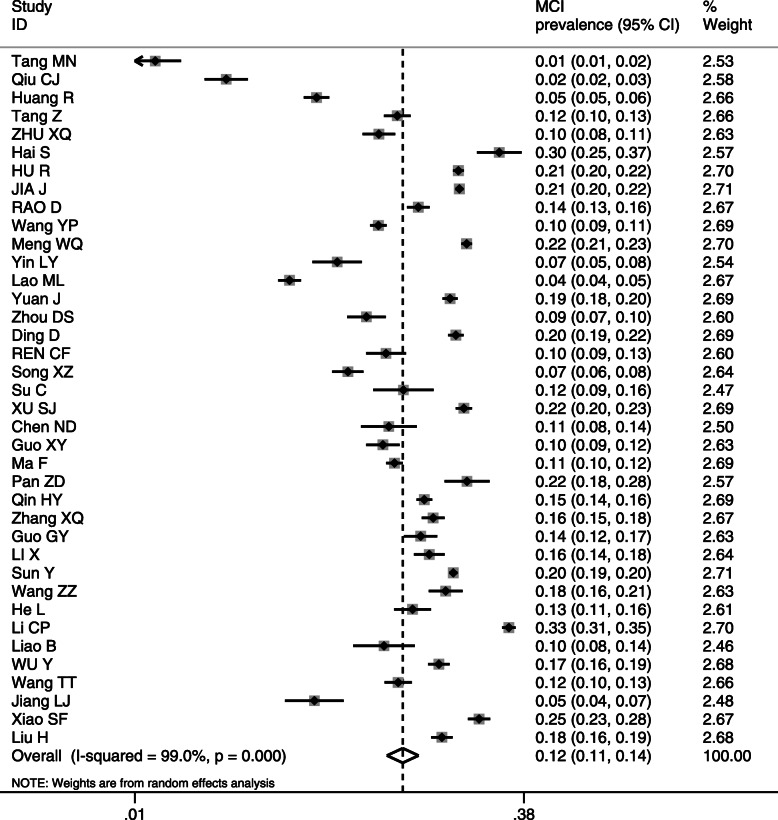
Prevalence of MCI

The meta-analysis of the 38 studies (*n* = 106,367) with a random-effect estimate resulted in a MCI prevalence of 12.2% [95% confidence interval (CI): 10.6, 14.2%] (Fig. 2).

**Fig. 3:**
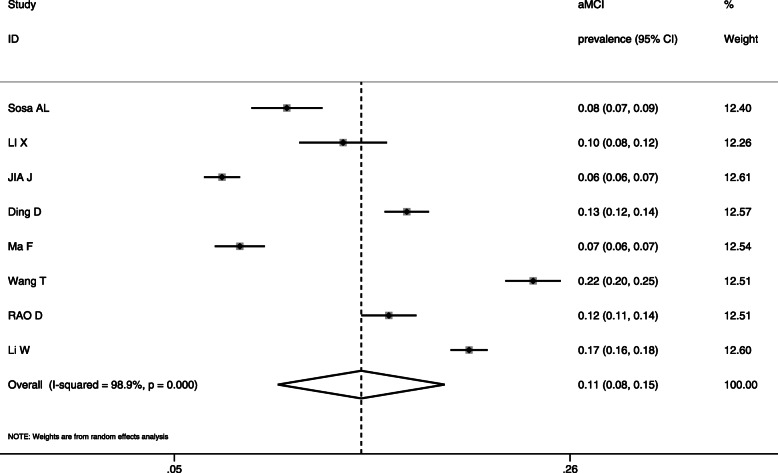
Prevalence of aMCI

The meta-analysis of the 8 studies (*n* = 27,613) generated a result of 10.9% prevalence of aMCI [95%CI: 7.7, 15.4%] (Fig. 3).

### Results of subgroup analyses

Heterogeneity of the subgroup analyses reduced significantly. The prevalence of MCI increased with age: 7.6% for 55–59 years; 9.5% for 60–69 years; 14.6% for 70–79 years; and 23.6% for 80 years and older. Women had a higher prevalence of MCI than men. Those who resided in rural areas, lived alone, and had lower educational attainments had higher MCI prevalence than others. The DSM-IV diagnostic criteria generated the highest MCI prevalence (13.5%), compared with 12.9% using the Petersen criteria and 10.3% using the NIA-AA diagnosis. The four studies [24, 42, 59, 60] conducted before 2005 reported significantly lower prevalence of MCI than those after 2005 (Table 4).

**Table 4:** Subgroup meta-analyses on the prevalence of MCI

Subgroup	Included studies	Study participants (sample size)	Random-effect Model		Heterogeneity	
			MCI Prevalence (95% CI)	p	I^**2**^	Ph
**Age (Years)**						
55–59	3	5951	0.076(0.025–0.226)	< 0.001	99.2%	0.922
60–69	24	23,095	0.095(0.074–0.121)	< 0.001	97.8%	0.356
70–79	25	22,902	0.146 (0.124–0.171)	< 0.001	96.6%	0.153
≥ 80	25	9397	0.236 (0.204–0.274)	< 0.001	93.5%	0.122
**Sex**						
Men	34	45,609	0.115 (0.097–0.136)	< 0.001	97.4%	0.233
Women	34	36,027	0.138 (0.117–0.163)	< 0.001	98.3%	0.224
**Residency**						
Urban	32	71,801	0.114 (0.098–0.132)	< 0.001	98.4%	0.174
Rural	12	25,137	0.136 (0.106–0.176)	< 0.001	98.8%	0.193
**Living status**						
With family	10	13,941	0.141 (0.110–0.182)	< 0.001	97.6%	0.157
alone	10	3518	0.182 (0.136–0.244)	< 0.001	95.0%	0.206
**Education attainment**						
< Primary school	18	10,974	0.172 (0.122–0.243)	< 0.001	98.3%	0.540
Primary school	18	14,502	0.120 (0.083–0.174)	< 0.001	98.5%	0.623
Middle school	21	11,367	0.091 (0.072–0.115)	< 0.001	94.3%	0.418
≥ High school	21	9568	0.063 (0.046–0.085)	< 0.001	94.2%	0.515
**Diagnostic criteria**						
Peterson	25	67,267	0.129 (0.107–0.154)	< 0.001	98.9%	0.209
DSM-IV	9	21,699	0.135 (0.097–0.188)	< 0.001	99.0%	0.257
NIA-AA	2	11,327	0.103 (0.029–0.369)	< 0.001	98.8%	0.833
**Study period**						
< 2005	4	15,857	0.037 (0.016–0.087)	< 0.001	99.2%	0.769
≥ 2005	30	90,510	0.141 (0.124–0.160)	< 0.001	98.6%	0.142

## Discussion

### Prevalence of MCI and aMCI

Overall, 12.2% of Chinese community-dwelling older adults have MCI. This result is consistent with findings of previous systematic reviews [21, 22] although they adopted much more broadened standards in terms of diagnostic confirmation and inclusion/exclusion criteria for included studies. If we exclude participants younger than 60 years in this systematic review, the result would be comparable to the MCI prevalence levels (12.7 to 14.7%) revealed in those systematic reviews. Similar levels of MCI prevalence were also reported in the 65 years and older populations in Greece [108] and Georgia [109]. Such a level is high compared to the studies conducted in other populations where more strict diagnostic criteria were adopted (e.g. neuropsychological scores at least 1.5 standard deviations below the adjusted norm). For example, a prevalence proportion of 5.3% for MCI was found in Finland in community residents aged between 60 and 76 years [110]. An Italian study found a prevalence proportion of 4.9% for MCI in community-dwelling residents older than 65 years [111]. It is likely that a higher percentage of older adults may have lived in aged care institutions in these developed nations. However, this does not offer a full explanation of the low prevalence of MCI. Lower levels of MCI prevalence were also found in some developing nations, such as 6.45% in Mexico [112] and 6.10% in Brazil [16] from community residents over 60 years despite the fact that most studies in these countries used the Petersen criteria, the same as the included studies in this current systematic review.

This study included participants aged between 55 and 59 years in the meta-analyses. To our knowledge, this is the first attempt to estimate MCI prevalence in people younger than 60 years. Indeed, there is a dramatic increase in MCI prevalence in the community residents older than 60 years as revealed in this study. However, 7.6% of those at the age between 55 and 59 years were still diagnosed with MCI. This indicates a potential benefit of starting MCI screening in this group of population. Many preventive care packages have been designed for people older than 55 years in community health services, which present an opportunity for introducing MCI screening services. But before such a policy is developed, robust studies into the cost benefits of such services are needed. Currently, there are few studies of MCI in people younger than 60 years. Only eight studies [24, 41, 44, 47, 51, 53, 59, 64] were identified in this systematic review. Nevertheless, the pathobiological process in the human brain happens decades before the onset of dementia [28] and MCI screening may reasonably start at the age of 55 years.

It is evident that aMCI is the predominant form of MCI in Chinese populations. This study estimated that 10.9% community-dwelling Chinese populations older than 55 years have aMCI, higher than those reported in most international studies [113, 114]. This study found that aMCI account for 66.5% of all MCI cases. Internationally, aMCI as a percentage of MCI ranges between 30 and 77%. The lowest prevalence of aMCI (2.4%) was reported in Mexico [112], while the highest (around 11%) was reported by the Mayo Clinic Study of Aging from Olmsted County, USA, residents between 70 and 89 years old [115].

### Factors associated with the prevalence of MCI and aMCI

High levels of heterogeneity are evident in the studies included in our meta-analyses. Many factors may have contributed to the variations of findings within individual studies. MCI prevalence varies by diagnostic tools, study settings and study periods. The lack of consensus in the definition of MCI has imposed serious challenges on previous reviews [25]. Different diagnostic confirmation tools can result in different MCI prevalence results [116]. The Petersen method [11] is based on four criteria: subjective memory complaint, objective memory disorder, normal functional activities, and absence of dementia. In contrast, the NIA-AA [103] allows inclusion of MRI imaging and cerebrospinal fluid tests as evidence, boosting the chance of MCI detection. However, our study shows that MCI prevalence is lower in the studies using NIA-AA compared to those applying the Petersen criteria. Such a contradiction may be associated with the fact that MRI imaging instruments and biomarker tests are optional and are likely to be ignored by many studies due to resource restrictions. Adding to the complexity is the use of screening as a first step to identify MCI patients. Variations in screening instruments and cut-off thresholds can lead to different results too [117]. MMSE [76] is the most commonly used cognitive screening tool worldwide, providing a comprehensive assessment on cognitive function in seven domains. However, the MMSE lacks sensitivity to detect MCI. While MoCA [77] meets the criteria with both high sensitivity and relatively high specificity in MCI detection (Sn = 81–97%; Sp = 60–86%) [118], it has been recommended as a preferred screening tool in MCI detection in primary care setting. It is believed that the variation of results across the study period can also be partly attributed to variations in screening diagnostic tools [119].

Differentiating between aMCI and naMCI may help address some of the above issues by offering greater clarity in selecting diagnostic and screening tools. But unfortunately, only a small percentage of studies chose to do so, perhaps because additional cognitive domains such as language, vision, and listening need to be assessed. It is important to note that the DSM-IV diagnostic confirmation method, used in 9 included studies in this systematic review, detects amnesic cognitive disorders and could underestimate the overall prevalence of MCI [120]. But it does not seem to be the case. This is likely to be a result of confounding effects of different screening tools.

Our sub-group meta-analyses revealed that MCI prevalence increases with age. Women, rural residents, and those who live alone and have low levels of education are likely to have higher MCI prevalence than others.

Aging has been reported as the most common risk factor for MCI [121]. Our study provides further evidence to support this argument. The prevalence of MCI in those aged between 70 and 79 years (14.6%) nearly doubles that of those aged between 55 and 59 years. The mechanism underling this age connection may be associated with increased oxidative stress and amyloidal accumulation in the brain [122].

In this study, we found that women are more likely to have MCI than men. This finding is consistent with results of previous systematic reviews on Chinese populations [21, 22]. This study showed, for the first time, that the same sex difference also exists in aMCI for Chinese populations, similar to that in other populations [123, 124]. Some researchers argued that hormone changes may explain the sex difference in MCI because there is evidence that hormone-replacement therapy can protect against dementia [125]. But the evidence is weak and indirect. There are studies reporting insignificant sex difference in MCI [126], or even higher prevalence of MCI in older adult men [115].

Socioeconomic disparities in MCI prevalence deserve increasing academic and policy attention. This systematic review confirmed that low levels of education can exacerbate the occurrence of cognitive impairment, including dementia as concluded in some other studies [127–129]. This is unlikely a result of screening or diagnostic bias as all neuropsychological tests have been corrected for education. Some researchers believe that education can enhance the brain’s ability to make efficient use of cognitive networks [130, 131]. Furthermore, those who live alone are more likely to have MCI. This may be associated with a lack of communication, anxiety, and depression [132]. The urban-rural difference in MCI prevalence may have some unique implications for China. Although it appears to be an international phenomenon with rural residents having higher MCI prevalence than their urban counterparts [133], possibly due to increased health risks and chronic conditions (such as diabetes and hypertension) [134–136], China’s dual welfare systems present some particular challenges for addressing the problem. Rural residents in mainland China usually have lower income, live in poorer housing conditions, receive less education, and enjoy lower levels of social and health entitlements compared with the urban ones. During the dramatic transition period with unprecedented economic development, a large proportion of young rural residents moved to urban centres for better education and job opportunities, leaving their older family members alone at home. A combination of these risk factors can expose rural older residents in a serious vulnerable position to cognitive impairment and dementia [137].

High levels of heterogeneity were observed for the pooled analysis of MCI and aMCI, as well as for the sub-group analysis of MCI. Although we adopted the recommended methods for handling the heterogeneity, it is noteworthy when applying the findings of this study. Indeed, previous studies reported an increase of MCI prevalence in China from 5% in 2000 to about 20% in 2014 [22]. This may be a result of several underlined reasons. We found in this study that cohort effect can explain about 21% of heterogeneity of the included studies in this systematic review. However, China has experienced dramatic socioeconomic transformation over the period. These include, but not limited to, prolonged life expectancy and arrival of an ageing society, increase in morbidity of chronic conditions such as diabetes and hypertension, and rapid advancement of medical technologies and medical care services. All of these can compound the prevalence of MCI. The true cohort effect can only be revealed through future studies using a method that can separate the effects of age, cohort, study period, and other confounding factors [138]. At this stage, the interpretation and application of the pooled results of this systematic review should be cautious. It is not unreasonable to anticipate a further increase in MCI prevalence as China continues its aging process. Local communities should consider the characteristics of their community residents in estimating local prevalence of MCI.

### Strengths and limitations

This study has several strengths. Firstly, this systematic review restricted studies to those of community-dwelling non-institutionalised residents. Very few, if any, Chinese people have received institutionalised care due to social, cultural and economic reasons. This enables better generalisability of findings to community populations. Secondly, this study included extensive literature searching for studies published in both English and Chinese languages. Thirdly, this study adopted more stringent inclusion and exclusion criteria to ensure high quality results. The analyses were based on confirmed, not suspected cases of MCI. Moreover, this systematic review involved a separate analysis on aMCI.

Despite the strengths, there are several limitations in this study. Lack of enough available data may account for the reasons why we had identified no more than one significant predictor in the meta-regression analysis. There is a shortage of studies into the subtypes of MCI, which prevented us from performing further subgroup analyses on aMCI prevalence. Only two studies [19, 20] included in the meta-analysis drew results from a nation-wide sample. The rest had participants from different regions. Significant socioeconomic disparities exist across regions in China. We found no study involving minority ethnicity groups. China has 56 ethnic groups. Further studies into these populations are needed. A national study using a unified protocol is preferred.

## Conclusion

This study shows that 12.2% of Chinese populations over 55 years have MCI and 10.9% have aMCI. MCI prevalence increases with age. Women, rural residents, and those who live alone and have low levels of education have higher MCI prevalence than others. The results also vary with diagnostic criteria and study periods. Increasing attention should be paid to regional disparities in future studies as socioeconomic disparities across regions continue to grow in China.

## Supplementary Information

**Additional file 1.**

**Additional file 2.**

## Data Availability

All data generated and analysed during this study are included in this manuscript and the supporting file.

## Abbreviations

ADL: Activity of Daily Living
ALT: Associative Learning Test
AMCI: Amnesic Mild Cognitive Impairment AVLT Auditory Verbal Learning Test
CAMCOG: Cambridge Cognitive Examination
CCAS: Chinese Cognitive Ability Scale
CDR: Clinical Dementia Rating Scale
CDT: Clock Drawing Test
CESD: The Center for Epidemiologic Studies Depression Scale
CSI-D: Community Screening Instrument for Dementia
DS: Digit Span
DSM-IV: the American Psychiatric Association’s Diagnostic and Statistical Manual of Mental Disorder 4th edition
FAQ: Functional Activities Questionnaire
GDS: Global Deterioration Scale
GMS: Geriatric Mental State
HAMD: Hamilton Depression Rating Scale
HIS: Hachinski Ischemic Index
IMCT: Information-Memory-Concentration test
MCI: Mild Cognitive Impairment
MMSE: Mini-Mental State Examination (MMSE)
MoCA: Montreal Cognitive Assessment
NIA-AA: National Institute on Aging Alzheimer’s Association (NIA-AA)
NPI-Q: Neuropsychiatric Inventory Questionnaire
QCST-E: Quick Cognitive Screening Scale
ROCFR: Rey-Osterrieth Complex Figure Recall Tests
SAS: Self-Rating Anxiety Scale
SCID: Structured Clinical Interview for DSM-IV
SDMT: Symbol Digit Modalities Test
STMT: Semantic Trail Making Test
VFT: Verbal Fluency Test
WAIS: Wechsler Adult Intelligence Scale
WHO-BCAI: World Health Organization-Battery of Cognitive Assessment Instrument
WHODAS-12: 12-item WHO Disability Assessment Schedule
WMS-R: Wechsler Memory Scale-Revised
