# Supplementary material for: Prevalence of mild cognitive impairment in community-dwelling Chinese populations aged over 55 years: a meta-analysis and systematic review
Source: BMC Geriatr. 2021 Jan 6;21:10. doi: 10.1186/s12877-020-01948-3 (PMC7789349; doi:10.1186/s12877-020-01948-3)
Supplement: Supplementary file 1 — Additional file 1. [file 12877_2020_1948_MOESM1_ESM.docx]

**Supporting information**

S1. Search strategy

S2. Data extraction

S3. Quality Assessment

**S1. Search strategy**

| **Source** |  | **Strategy** |
| --- | --- | --- |
| 1 | **Pubmed** | 1．"Cognitive Dysfunction/epidemiology"[Mesh]  2．”mild cognitive impairment”[Title/Abstract]  3. MCI[Title/Abstract]  4. prevalence[Title/Abstract]  5. epidemiology[Title/Abstract]  6. frequency[Title/Abstract]  7. occurrence[Title/Abstract]  8. Affiliation=China  9. Chinese or China or province  10. 1 OR 2 OR 3  11. 4 OR 5 OR 6 OR 7  12. 8 OR 9  13. 10 AND 11 AND 12 |
|  | **Result** | 120 |
| 2 | **Embase**  **Result** | 1. Exp mild cognitive impairment 2. ("mild cognitive impairment" or MCI).mp. limit to (abstracts and human) 3. (prevalence or incidence or epidemiology or frequency or occurrence).mp. limit to (abstracts and human) 4. (China or Chinese).mp. limit to (abstracts and human) 5. 1 OR 2 6. 3 AND 4 AND 5   31 |
| 3 | **PsycINFO**  **Result** | 1. "mild cognitive impairment" or MCI.mp[mp=title, abstract, heading word, table of contents, key concepts, original title, tests & measures, mesh] 2. mild cognitive impairment.mp. 3. exp Cognitive Impairment/ 4. 1 OR 2 OR 3 5. exp Epidemiology/ 6. (Prevalence or incidence or epidemiology or frequency or occurrence).mp. 7. 5 0R 6 8. (China or Chinese).mp. 9. 4 AND 7 AND 8   106 |
| 4 | **Webofscience**  **Result** | 1. (TS=MCI or TS=mild cognitive impairment) AND language: (English or Chinese) AND document types: (Article) 2. (TI=mild cognitive impairment or TI=MCI) AND language: (English or Chinese) AND document types: (Article) 3. 1 OR 2 4. (TS=prevalence or TS=epidemiology) AND language: (English or Chinese) AND document types: (Article) 5. (TI=Prevalence or TI=incidence or TI=epidemiology or TI=frequency or TI=occurrence) AND language: (English or Chinese) AND document types: (Article) 6. 4 OR 5 7. (AD=China or AD=Chinese) AND language: (English or Chinese) AND document types: (Article) 8. (CU=China or CU=Chinese) AND language: (English or Chinese) AND document types: (Article) 9. 7 OR 8 10. 3 AND 6 AND 9   122 |
| 5 | **CINAHL**  **Result** | 1. AB prevalence or epidemiology or frequency or occurrence 2. (MM "Prevalence") OR (MH "Epidemiology+") OR (MH "Surveys+") OR (MM "Cross Sectional Studies") 3. AB mild cognitive impairment OR AB MCI 4. (MH "Cognition Disorders+/DI/EP/SU") 5. China OR Chinese 6. 1 OR 2 7. 3 OR 4 8. 5 AND 7   86 |
| 6 | **WanFang**  **Result** | 1. 主题:轻度认知功能障碍 2. 题名:轻度认知功能障碍 3. 摘要:轻度认知功能障碍 4. 关键词:轻度认知功能障碍 5. 1 OR 2 OR 3 OR 4 6. 主题:患病率 7. 题名:患病率 8. 摘要:患病率 9. 关键词:患病率 10. 6 OR 7 OR 8 OR 9 11. 5 AND 10   446 |
| 7 | **Cnki**  **Result** | 1. SU='轻度认知功能障碍' 2. TI='轻度认知功能障碍' 3. KY='轻度认知功能障碍' 4. AB='轻度认知功能障碍' 5. 1 OR 2 OR 3 OR 4 6. SU=‘患病率’ 7. TI=‘患病率’ 8. KY=‘患病率’ 9. AB=‘患病率’ 10. 6 OR 7 OR 8 OR 9 11. 5 AND 10   1444 |
| 8 | **Sinomed**  **Result** | 1. Exp"患病率"[中文标题:智能]) 2. Exp "患病率"[摘要:智能]) 3. Exp "患病率"[关键词:智能]) 4. Exp "患病率"[不加权:扩展]) 5. Exp "患病率"[特征词] 6. 1 OR 2 OR 3 OR 4 OR 5 7. Exp "轻度认知障碍"[中文标题:智能] 8. Exp "轻度认知障碍"[摘要:智能] 9. Exp "轻度认知障碍"[关键词:智能] 10. Exp "轻度认知障碍"[特征词] 11. Exp "轻度认知障碍"[特征词] 12. 7 OR 8 OR 9 OR 10 OR 11   490 |
| 9 | **CQVIP**  **Result** | 1. M=轻度认知障碍 2. R=轻度认知障碍 3. K=轻度认知障碍 4. 1 OR 2 OR 3 5. M=患病率 6. R=患病率 7. K=患病率 8. 5 OR 6 OR 7 9. 4 AND 8   88 |
|  |  |  |

**S2. Data extraction**

| **ID** | **Author** | **Year** | **Location** | **Exclusion Criteria** |
| --- | --- | --- | --- | --- |
| 1 | Ailikemu.Abudouwallke | 2011 | Xinjiang | According to exclusion criteria 5 |
| 2 | Bai Jiao-jiao | 2003 | Shanghai | According to exclusion criteria 5 |
| 3 | Chen Chang-xiang | 2008 | Tianjin | According to exclusion criteria 3 |
| 4 | Chen chen | 2018 | Sichuan | According to exclusion criteria 5 |
| 5 | Chen Ding-hua | 2013 | Shanghai | According to exclusion criteria 7 |
| 6 | Chen juan | 2014 | Beijing | According to exclusion criteria 2 |
| 7 | Chen nian-dong | 2014 | Jiangsu | According to exclusion criteria 5 |
| 8 | Chen qing-you | 2015 | Heilongjiang | According to exclusion criteria 5 |
| 9 | Chen xue-ping | 2009 | Zhejiang | According to exclusion criteria 5 |
| 10 | Chen Yu-min | 2015 | Shanghai | According to exclusion criteria 7 |
| 11 | Deng qian | 2013 | national | According to exclusion criteria 3 |
| 12 | Deng yue-xian | 2014 | Hubei | According to exclusion criteria 5 |
| 13 | Ding ding | 2012 | Shanghai | According to exclusion criteria 7 |
| 14 | Ding Li | 2013 | Ningxia | According to exclusion criteria 5 |
| 15 | Ding Shu-ping | 2009 | 22 Province | According to exclusion criteria 5 |
| 16 | Fan jing-bo | 2014 | Shan dong | According to exclusion criteria 5 |
| 17 | Fang Gui-zhen | 2009 | zhejiang | According to exclusion criteria 2,5 |
| 18 | Fang hong | 2015 | Shanghai | According to exclusion criteria 5 |
| 19 | Gao Li-wen | 2011 | Jiangsu | According to exclusion criteria 5 |
| 20 | Ge gai-zhen | 2009 | Beijing | According to exclusion criteria 6,7 |
| 21 | Gu bian-bian | 2014 | Anhui | According to exclusion criteria 5 |
| 22 | Gu xiao-lei | 2008 | Shanghai | According to exclusion criteria 5 |
| 23 | Guan shao-chen | 2008 | Beijing | According to exclusion criteria 7 |
| 24 | Hai shan | 2010 | Sichuan | According to exclusion criteria,3 |
| 25 | Han rui | 2017 | Beijing | According to exclusion criteria 3 |
| 26 | He Xiao-yan | 2013 | Xinjiang | According to exclusion criteria 3,5 |
| 27 | Hong zhen | 2000 | Shanghai | According to exclusion criteria 3 |
| 28 | HU Riletemue | 2012 | Inher Mongolia | According to exclusion criteria 5 |
| 29 | Huang Li-quan | 2007 | Shen zhen | According to exclusion criteria 5 |
| 30 | Huang Wen-yong | 2008 | Guiyang | According to exclusion criteria 5 |
| 31 | Huang Wu-quan | 2019 | Shanghai | According to exclusion criteria 5 |
| 32 | Huo dong-hong | 2000 | Shanxi | According to exclusion criteria 3,5 |
| 33 | Ji nan-nan | 2017 | Xinjiang | According to exclusion criteria 5 |
| 34 | Jia hai-lin | 2011 | Hebei | According to exclusion criteria 6 |
| 35 | jiang hai | 2013 | Jiang su | According to exclusion criteria 5 |
| 36 | Jiang hu | 2019 | Guangxi | According to exclusion criteria 5 |
| 37 | Lei ming-yu | 2008 | Guizhou | According to exclusion criteria 5 |
| 38 | Li Jing | 2013 | Shandong | According to exclusion criteria 5 |
| 39 | Li ming-qiu | 2017 | Hubei | According to exclusion criteria 2,5 |
| 40 | Li peng | 2019 | Shanghai | According to exclusion criteria 2,5 |
| 41 | Li Zhi-bin | 2013 | Beijing | According to exclusion criteria 5 |
| 42 | Li Zhi-wu | 2013 | Beijing | According to exclusion criteria 5 |
| 43 | Liang Wei-ping | 2008 | Shan xi | According to exclusion criteria 5 |
| 44 | Liu Bin | 2005 | Guangzhou | According to exclusion criteria 5 |
| 45 | Liu hong-yan | 2018 | Anhui | According to exclusion criteria 5 |
| 46 | Liu jia-lu | 2019 | Shanghai | According to exclusion criteria 5 |
| 47 | Liu Miao | 2018 | Beijing | According to exclusion criteria 5 |
| 48 | Liu xiao-jun | 2018 | Hubei | According to exclusion criteria 5 |
| 49 | Lu Hui | 2019 | Tianjin | According to exclusion criteria 3,7 |
| 50 | Lu yan-hui | 2011 | Beijing | According to exclusion criteria 3 |
| 51 | Luo Jian-fen | 2015 | Shanghai | According to exclusion criteria 7 |
| 52 | Luo xiong | 2015 | Guangzhou | According to exclusion criteria 7 |
| 53 | Lv Quan-jun | 2016 | Henan | According to exclusion criteria 5 |
| 54 | Ma chao | 2013 | Beijing | According to exclusion criteria 7 |
| 55 | Ma min | 2017 | Jilin | According to exclusion criteria 7 |
| 56 | Meng yuan-yuan | 2010 | Shandong | According to exclusion criteria 2 |
| 57 | Nie Hong-wei | 2011 | Suzhou | According to exclusion criteria 5 |
| 58 | Qiu Yue-hong | 2018 | Guangzhou | According to inclusion criteria 4 |
| 59 | Sha yi | 2011 | Xinjiang | According to exclusion criteria 5 |
| 60 | Shen ying | 2011 | Guangzhou | According to exclusion criteria 7 |
| 61 | Shi Zhi-hong | 2013 | Tianjin | According to exclusion criteria 3 |
| 62 | Song mei | 2014 | He bei | According to exclusion criteria 5 |
| 63 | Song ya-jun | 2013 | Beijing | According to exclusion criteria 3 |
| 64 | Su chan | 2013 | guangzhou | According to exclusion criteria 5 |
| 65 | Su Ning | 2019 | Shanghai | According to exclusion criteria 7 |
| 66 | Su Xiang-ni | 2013 | Shan Xi | According to exclusion criteria 5 |
| 67 | Tang Ya-qin | 2016 | Hu Bei | According to exclusion criteria 5 |
| 68 | Tao Shu-li | 2019 | Beijing | According to exclusion criteria 2 |
| 69 | Tao Xue-qin | 2014 | Jiangxi | According to exclusion criteria 5 |
| 70 | Tong Jiu-fen | 2013 | Hebei | According to exclusion criteria 6 |
| 71 | Wan Chang-min | 2018 | Hunan | According to exclusion criteria 2,3 |
| 72 | Wang Ai-min | 2016 | Hunan | According to exclusion criteria 5 |
| 73 | Wang Feng | 2010 | Shandong | According to exclusion criteria 6 |
| 74 | Wang Lu | 2011 | Shandong | According to exclusion criteria 2.3 |
| 75 | Wang ping | 2007 | Shanxi | According to exclusion criteria 3 |
| 76 | Wang Qian-qian | 2012 | Tianjin | According to exclusion criteria 5 |
| 77 | Wang xing-wei | 2014 | Jiangsu | According to exclusion criteria 2 |
| 78 | Wang yong-bin | 2013 | Shanghai | According to exclusion criteria 7 |
| 79 | Wang Yu-mei | 2015 | Hebei | According to exclusion criteria 2 |
| 80 | Wang zhi-zhong | 2016 | Ningxia | According to exclusion criteria 6,7 |
| 81 | Wang Zhui-qin | 2013 | zhejiang | According to exclusion criteria 5 |
| 82 | Wen Fang | 2012 | Sichuan | According to exclusion criteria 2,5 |
| 83 | WU bin | 2012 | Shanxi | According to exclusion criteria 5 |
| 84 | Wu ye-guang | 2005 | Guangxi | According to exclusion criteria 5 |
| 85 | Xiang Jie | 2009 | Jiangsu | According to exclusion criteria 5 |
| 86 | Xiao rui-jiang | 2013 | guangzhou | According to exclusion criteria 3 |
| 87 | Xiaojun | 2015 | Sichuang | According to exclusion criteria 1 |
| 88 | Xie Hen-ge | 2003 | Shan Dong | According to exclusion criteria 2,5 |
| 89 | Xiong Yin | 2013 | Tianjin | According to exclusion criteria 5 |
| 90 | Xu Ming-yin | 2001 | Shanghai | According to exclusion criteria 5 |
| 91 | Xu wen-xiu | 215 | Guizhou | According to exclusion criteria 3 |
| 92 | Xue Jun | 2012 | zhejiang | According to exclusion criteria 2,5 |
| 93 | Xue Zhi-ling | 2010 | Shanxi | According to exclusion criteria 2,5 |
| 94 | Yan Zou | 2014 | Chongqing | According to exclusion criteria 2 |
| 95 | Yang Fan | 2010 | Hu Bei | According to exclusion criteria 5 |
| 96 | Yang feng-jiao | 2010 | Hu nan | According to exclusion criteria 5 |
| 97 | Yang Hong-qing | 2018 | FU JIAN | According to exclusion criteria 3,5 |
| 98 | Yang jing-yuan | 2008 | Guizhou | According to exclusion criteria 5 |
| 99 | Yang yu-huan | 2017 | Hubei | According to exclusion criteria 5 |
| 100 | Yao qian | 2016 | Shanghai | According to exclusion criteria 6 |
| 101 | Yi Gang | 2010 | Sichuan | According to exclusion criteria 5 |
| 102 | Yi Yi-shaya | 2011 | Xinjiang | According to exclusion criteria 5 |
| 103 | Yin Shu-qin | 2011 | zhejiang | According to exclusion criteria 5 |
| 104 | Yu Elena S.H. | 1989 | Shanghai | According to exclusion criteria 3 |
| 105 | Yuan lu | 2017 | Guizhou | According to exclusion criteria 2 |
| 106 | Zhai jing-guo | 2004 | Shandong | According to exclusion criteria 3 |
| 107 | Zhang Cai-pin | 2010 | Shan Xi | According to exclusion criteria 5 |
| 108 | Zhang Hai-yan | 2014 | Tianjin | According to exclusion criteria 5 |
| 109 | Zhang Jian-an | 2016 | Jiangsu | According to exclusion criteria 5 |
| 110 | Zhang Pin-hua | 2014 | Sichuang | According to exclusion criteria 3 |
| 111 | Zhang xue-fei | 2014 | zhejiang | According to exclusion criteria 3,5 |
| 112 | Zhang Xue-lin | 2008 | zhejiang | According to exclusion criteria 2,5 |
| 113 | Zhang yao-dong | 2011 | Jiang su | According to exclusion criteria 5 |
| 114 | Zhang ying | 2014 | Tianjin | According to exclusion criteria 3 |
| 115 | Zhang ying-xin | 2016 | Chongqing | According to exclusion criteria 3 |
| 116 | Zhang ying-yi | 2002 | Chongqing | According to exclusion criteria 5 |
| 117 | Zhang yuan | 2018 | Shanghai | According to exclusion criteria 5 |
| 118 | Zhao Chun-shan | 2015 | jI lin | According to exclusion criteria 5 |
| 119 | Zhao Jian-gang | 2015 | Tianjin | According to exclusion criteria 5 |
| 120 | Zhao Yu-jin | 2018 | Tianjin | According to exclusion criteria 4 |
| 121 | Zhou Dong-mei | 2016 | Xinjiang | According to exclusion criteria 5 |
| 122 | Zhou li-ye | 2010 | Shanxi | According to exclusion criteria 5 |
| 123 | Zhu Ai-qun | 2015 | Shanghai | According to exclusion criteria 5 |
| 124 | Zhu Hong-xia | 2009 | Shanghai | According to exclusion criteria 2 |
| 125 | Zhu xin-hong | 2018 | zhejiang | According to exclusion criteria 5 |
| 126 | Zhu ya-ping | 2013 | Zhejiang | According to exclusion criteria 5 |
| 127 | Zhuo Chuan-jun | 2012 | Beijing | According to exclusion criteria 6 |

**Exclusion criteria**

1. Letters, commentaries, reviews, case-control studies, experimental studies, clinical trials

2. Sampling from institutions or samples restricted to those with special characteristics such as disease condition (e.g. Parkinson disease, depression, stroke), occupation, internal migration, insurance, and literacy.

3. Cognitive decline in general as a study outcome

4. MCI screening without diagnostic confirmation

5. Original data is unavailable

6. Data results has been used elsewhere

**S3. Quality Assessment**

| **ID** | **Study** | **External validity** | | | | **Internal validity** | | | | | | **Overall** |
| --- | --- | --- | --- | --- | --- | --- | --- | --- | --- | --- | --- | --- |
|  |  | 1 | 2 | 3 | 4 | 5 | 6 | 7 | 8 | 9 | 10 |  |
| 1 | Chen ND, 2012 | M | M | M | M | L | L | M | L | M | M | M |
| 2 | Ding D, 2015 | M | M | L | L | L | L | L | L | M | L | L |
| 3 | Guo GY, 2013 | M | M | M | M | L | L | M | L | M | M | M |
| 4 | GuoXY, 2013 | M | M | L | M | L | L | M | L | L | M | M |
| 5 | Hai S, 2011 | H | M | L | M | L | L | M | L | L | M | M |
| 6 | He L, 2015 | M | M | L | L | L | L | M | L | L | M | M |
| 7 | Hu R, 2012 | M | M | M | L | L | M | M | L | M | M | M |
| 8 | Huang R, 2008 | M | L | L | L | L | L | L | L | L | M | L |
| 9 | JIA J, 2013 | L | L | L | L | L | L | L | L | M | L | L |
| 10 | Jiang LJ, 2017 | M | M | M | M | L | L | M | L | M | M | M |
| 11 | Lao ML, 2011 | M | M | L | M | L | L | M | L | M | M | M |
| 12 | Li CP, 2014 | M | M | L | L | L | L | M | L | M | M | M |
| 13 | Li X, 2013 | M | M | L | M | L | L | M | L | M | L | M |
| 14 | Li W, 2020 | M | M | L | L | L | L | L | L | M | M | L |
| 15 | Liao B, 2012 | M | M | L | M | L | L | M | L | M | M | M |
| 16 | Liu H, 2018 | M | M | L | L | L | M | M | L | L | M | M |
| 17 | Ma F, 2016 | M | M | L | L | L | L | L | L | M | L | L |
| 18 | Meng WQ, 2010 | M | M | L | M | L | L | M | L | M | M | M |
| 19 | Pan HY, 2012 | M | M | M | L | L | H | L | L | L | M | H |
| 20 | Pan ZD, 2012 | M | M | L | L | L | L | M | L | L | M | M |
| 21 | Peng Z, 2019 | M | M | L | M | L | H | M | L | L | M | H |
| 22 | Qin HY, 2014 | M | M | L | L | L | L | L | L | L | M | L |
| 23 | Qiu CJ, 2003 | M | L | L | M | L | M | H | L | L | M | M |
| 24 | Rao D, 2018 | M | L | L | L | L | L | M | L | L | L | L |
| 25 | Ren CF, 2013 | M | M | L | M | L | L | L | L | M | M | M |
| 26 | Song XZ, 2012 | M | M | L | L | L | M | M | L | M | M | M |
| 27 | Sosa AL, 2012 | L | M | L | M | L | L | M | L | L | M | M |
| 28 | Su C, 2013 | M | M | M | M | L | L | M | L | M | M | M |
| 29 | Sun Y, 2014 | M | M | L | L | L | M | M | L | M | M | M |
| 30 | Tang Z, 2007 | M | M | L | L | L | L | M | L | M | M | M |
| 31 | Tang MN, 2000 | M | M | L | L | L | M | H | L | M | M | M |
| 32 | Wang T, 2017 | M | M | L | L | L | M | M | L | M | M | M |
| 33 | Wang TT, 2017 | M | M | L | L | L | L | L | L | L | M | L |
| 34 | Wang YP, 2011 | M | M | M | L | L | L | M | L | M | M | M |
| 35 | Wang ZZ, 2013 | M | M | H | L | L | L | M | L | M | M | M |
| 36 | Wu L, 2016 | M | M | L | L | L | H | M | L | M | M | H |
| 37 | Wu Y, 2017 | M | L | L | M | L | L | M | L | M | M | M |
| 38 | Xiao SF, 2016 | M | M | L | L | L | L | M | L | L | M | L |
| 39 | Xu SJ, 2014 | M | M | L | L | L | L | M | L | L | M | M |
| 40 | Yin LY, 2010 | M | L | L | M | L | L | M | L | L | M | M |
| 41 | Yuan J, 2013 | M | M | L | M | L | L | M | L | L | M | M |
| 42 | Zhang XQ, 2014 | M | M | L | L | L | L | L | L | H | M | L |
| 43 | Zhong SY, 2018 | M | M | L | M | L | H | M | L | M | M | H |
| 44 | Zhou DS, 2011 | M | M | L | M | L | L | L | L | M | M | M |
| 45 | Zhu XQ, 2009 | M | M | L | M | L | L | L | L | M | M | M |

**Items notes:** L—low risk; M-middle risk; H-high risk

**External validity**

1. Was the study’s target population a close representation of the national population in relation to relevant variables?
2. Was the sampling frame a true or close representation of the target population?
3. Was some form of random selection used to select the sample, OR was a census undertaken?
4. Was the likelihood of non response bias minimal?

**Internal validity**

1. Were data collected directly from the subjects (as opposed to a proxy)?
2. Was an acceptable case definition used in the study?
3. Was the study instrument that measured the parameter of interest shown to have validity and reliability?
4. Was the same mode of data collection used for all subjects?
5. Was the length of the shortest prevalence period for the parameter of interest appropriate?
6. Were the numerator(s) and denominator(s) for the parameter of interest appropriate?
